# Immobilization of *Rhodococcus* by encapsulation and entrapment: a green solution to bitter citrus by-products

**DOI:** 10.1007/s00253-023-12724-9

**Published:** 2023-08-24

**Authors:** María C. Pilar-Izquierdo, María López-Fouz, Natividad Ortega, María D. Busto

**Affiliations:** Department of Biotechnology and Food Science, University of Burgos, Plaza Misael Bañuelos, S/N, 09001 Burgos, Spain

**Keywords:** Cell immobilization, Ca-alginate hollow beads, PVA/PEG cryogels, *Rhodococcus*, Debittering, Citrus by-products

## Abstract

**Abstract:**

Debittering of citrus by-products is required to obtain value-added compounds for application in the food industry (e.g., dietary fiber, bioactive compounds). In this work, the immobilization of *Rhodococcus fascians* cells by encapsulation in Ca-alginate hollow beads and entrapment in poly(vinyl alcohol)/polyethylene glycol (PVA/PEG) cryogels was studied as an alternative to chemical treatments for degrading the bitter compound limonin. Previously, the *Rhodococcus* strain was adapted using orange peel extract to increase its tolerance to limonoids. The optimal conditions for the encapsulation of microbial cells were 2% Na-alginate, 4% CaCl_2_, 4% carboxymethylcellulose (CMC), and a microbial load of 0.6 OD_600_ (optical density at 600 nm). For immobilization by entrapment, the optimal conditions were 8% PVA, 8% PEG, and 0.6 OD_600_ microbial load. Immobilization by entrapment protected microbial cells better than encapsulation against the citrus medium stress conditions (acid pH and composition). Thus, under optimal immobilization conditions, limonin degradation was 32 and 28% for immobilization in PVA/PEG gels and in hollow beads, respectively, in synthetic juice (pH 3) after 72 h at 25 °C. Finally, the microbial cells entrapped in the cryogels showed a higher operational stability in orange juice than the encapsulated cells, with four consecutive cycles of reuse (runs of 24 h at 25 °C).

**Key points:**

• *Increased tolerance to limonoids by adapting R. fascians with citrus by-products.*

• *Entrapment provided cells with favorable microenvironment for debittering at acid pH.*

• *Cryogel-immobilized cells showed the highest limonin degradation in citrus products.*

## Introduction

The application of immobilized microorganisms is gaining importance in areas such as bioremediation, biocatalysis, and industrial processing (Krivoruchko et al. 2019). The advantages of immobilization include increased productivity, cell reuse, and enhanced stability, the latter due to the protective effect of immobilization (Lapponi et al. 2022). However, immobilization also has its disadvantages, as it is not suitable for every biocatalyst, increases the production cost, and could affect the process performance (e.g., diffusion limitations) (Krasňan et al. 2016). Therefore, it is necessary to find the most suitable immobilization methodology for each specific application.

Immobilization processes for industrial biotransformation should be relatively simple, cost-effective, and prove active biocatalyst with substantial stability (Sheldon 2007). Various methods and carriers for immobilizing cells have been described in the literature, depending largely their choice on the final application. Cell immobilization methods include entrapment, encapsulation, adsorption, or covalent binding, with entrapment being the preferred method (Lapponi et al. 2022). Carriers for cell immobilization must be biocompatible, insoluble, nontoxic, inexpensive, and not biodegradable by the biomass (Bouabidi et al. 2019). A variety of carriers have been used for cell immobilization, including natural (e.g., agar, alginate) and synthetic polymers (e.g., polyacrylamide, polyvinyl) (Katzbauer et al. 1995). A commonly used method for the immobilization of biocatalysts is the encapsulation in Ca-alginate hollow beads (Dembczynski and Jankowski 2002; Chai et al. 2004; Ruiz et al. 2018), which is a simple, biocompatible, and low-cost method, among other advantages (Chai et al. 2004). Encapsulation involves the retention of cells in a liquid core surrounded by a semipermeable membrane (Dembczynski and Jankowski 2002) in the form of spheres (hollow beads). Another widely reported method of cell immobilization is the entrapment in cryogels based on PVA (Lozinsky and Plieva 1998; Szczęsna-Antczak et al. 2004; du Toit and Pott 2020), a biocompatible, nontoxic, and inexpensive polymer. In contrast to encapsulation, in the entrapment, the cells are retained by the cryogel-forming event; thus, they are dispersed within the polymeric matrix. PVA cryogels possess interesting properties as carriers for cell immobilization, such as chemical and mechanical stability, and resistance to biological degradation (Lozinsky 2014). Moreover, cryogels prepared through freeze/thaw cycle result in a highly porous structure, which is fundamentally important to facilitate the diffusion of molecules towards the immobilized cells. In addition, the properties of these cryogels can be modified by adding polymers such as polyethylene glycol (PEG) to the PVA solution (Falqi et al. 2018).

Citrus processing generates large amounts of by-products that can be harmful to the environment if not disposed of properly (Suri et al. 2022). The main by-product generated is citrus peel, which can account for up to 60% of the total waste (Chavan et al. 2018). Strategies for citrus by-products valorisation have increasing in the last years, as these wastes contain potentially valuable compounds (e.g., dietary fiber, bioactive compounds) that can be used as ingredients in the food industry (Nieto et al. 2021; Russo et al. 2021; Panwar et al. 2021). However, sensory properties such as bitterness due to limonin, the bitter principle of citrus fruits, may limit their use in the food industry. Studies using chemical methods, such as alkali treatment, have been described for the debittering of citrus by-products (Caggia et al. 2020; Singla et al. 2021). In this way, biotechnological treatments using microorganisms may be a more sustainable option compared to the use of chemicals. Bacteria of the genus *Rhodoccocus* are good candidates for this purpose due to their metabolic flexibility and resistance to stress conditions (Cappelletti et al. 2020; Pátek et al. 2021). Specifically, *Rhodococcus fascians* NRRL-B-15096 synthesizes enzymes that can convert bitter limonoids to non-bitter compounds (Hasegawa and King 1983; Cánovas et al. 1998). Although studies on limonin degradation by immobilized *R. fascians* have been described (Martínez-Madrid et al. 1989; Iborra et al. 1994; Cánovas et al. 1998), to date the reported methods have neither been satisfactory nor suitable for application. These results could be partially explained by the immobilization method used and the characteristics of the application medium itself, which may affect the survival and the metabolism of the microorganisms. In fact, it had been reported the antimicrobial activity of some compounds present in citrus, such as limonoids, being the Gram-positive, as *R. fascians*, the more sensitive bacteria (Kowalska et al. 2017; Oikeh et al. 2016). Moreover, the acidic pH of the citrus and by-products (Ruiz and Flotats 2014) could affect the performance of the process.

In this work, we optimized the immobilization of *R. fascians* NRRL-B-15096 by encapsulation in Ca-alginate hollow beads and entrapment in PVA/PEG cryogels, and compared its potential to degrade limonin. The microbial strain was first conditioned to increase its tolerance to limonoids using orange peel extract. Furthermore, the operational stability of the immobilized bacterial cells was investigated at pH 7 (Martínez Madrid medium) and at acid pH of 3.5 (orange juice).

## Materials and methods

### Materials and bacterial strain

Carboxymethylcellulose (CMC) sodium salt low viscosity and sodium alginate were supplied by Sigma-Aldrich (Merck KGaA, Darmstadt, Germany). Poly (vinyl alcohol) (PVA) (number average molecular weight, *M*_*n*_, of 72,000; degree of polymerization of 1600; saponification value ˃97.5%) and polyethylene glycol 600 (PEG) were purchased from Fluka (Fisher Scientific, Madrid, Spain). All other chemicals and reagents were of analytical grade.

Limonin (84% purity) was extracted from citrus seeds following the procedure described by Pifferi et al. (1993).

*Rhodococcus fascians* NRRL-B-15096 was obtained from the Spanish Official Culture Collection (Valencia, Spain). The bacterial strain was precultured on agar slants at 25 °C for 48 h in Martínez Madrid (MM) medium (Martinez-Madrid et al. 1989) and stored at 4 °C for further use.

### Tolerance and adaptation of *R. fascians* to limonoids

The tolerance of *R. fascians* to compounds presents in citrus by-products, such as limonoids, was tested by growing the microorganism in MM medium without fructose, supplemented with different concentrations of an orange peel extract. The extract was obtained following the method reported by Larrauri et al. (1996) and modified by Meza (2003). Briefly, orange peel (from *Valencia-late* oranges) was oven dried at 50 °C for 5 days, then ground, sieved (< 0.1 mm), and stored in a desiccator at room temperature prior to use. The ground orange peel was mixed to obtain the extract and homogenized with distilled water (proportion 1/7, w/v) and the pH was adjusted to 5.5 with 0.2 M NaOH. Then, the mixture was maintained in a water-bath at 90 °C for 5 min followed by hot filtration (Whatman nº1) at low pressure.

The culture media for the tolerance study were prepared in an MM culture medium without fructose and 5, 10, 20, 40, 60, 80, and 100% (v/v) of orange peel extract. Media pH was set at 7.0 with 1 M NaOH before sterilization. One hundred-milliliter Erlenmeyer flasks, each with 20 mL of the culture medium, were supplemented with 2% of inoculum precultured in MM medium at 25 °C, 150 rpm for 48 h, with an optical density at 600 nm (OD_600_) of 1.2. The flasks were incubated at 25 °C, 150 rpm for 48 h, and bacterial growth was determined by measuring the OD_600_ (Unicam 5625).

The adaptation procedure of *R. fascians* was based on the batch serial cultures with increasing concentrations of orange peel extract (5 to 100%, v/v). Afterwards, the microorganism was maintained by growing on agar slant at 25 °C for 48 h in MM medium with 60 µg mL^−1^ of limonin as the only carbon source.

### Immobilization of *R. fascians*

To immobilize the microbial cells, a bacterial inoculum was prepared by growing *R. fascians* in MM medium with limonin (60 µg mL^−1^) at 25 °C, 150 rpm for 18 h. Thereupon, the OD_600_ of the culture was measured and dilutions of 0.1% peptone water were added to reach OD_600_ of 0.2, 0.4, 0.6, 0.9, and 1.2. Thirty milliliters of each dilution was centrifuged at 7,800 g for 15 min. The resulting bacterial pellet was resuspended in 1.5 mL of 0.1% peptone water, constituting the bacterial inoculum.

#### *Encapsulation of* R. fascians *in alginate hollow beads*

The bacterial cells were encapsulated in Ca-alginate hollow beads adapting the method reported by Patel et al. (2000) and modified by Pilar-Izquierdo et al. (2013). The bacterial inoculum (1.5 mL) was mixed with 8.5 mL of CMC solution (4%, w/v) containing CaCl_2_ (between 2 and 4%, w/v). The mixture was dropped through a peristaltic pump into 300 mL of 2% sodium alginate and gently stirred in a 1L beaker. After 10 min of gelation, the alginate solution was decanted, and the resulting hollow beads were washed three times with deionized water (300 mL). Then, the beads were transferred into 2% (w/v) CaCl_2_, stirred, and left to harden for 20 min. Finally, the hollow beads were washed with 0.06% (v/v) sodium hypochlorite, rinsed with sterilized distilled water, and stored in 2% (w/v) CaCl_2_ at 4 °C until use.

#### Entrapment of microbial cells in PVA/PEG cryogels

The microbial strain was entrapped in PVA/PEG cryogels by a freeze-thawing process adapting the method reported by Ariga et al. (1993). The immobilization solution was prepared by dissolving PVA (7 or 8%) and PEG (8 or 10%) in 18.5 mL of 67 mM phosphate buffer (pH 8.0) by autoclaving for 20 min at 121 °C, cooled to room temperature and mixed with 1.5 mL of the bacterial inoculum. The immobilization solution was dropped into liquid nitrogen, whereupon the PVA/PEG beads were formed instantly. After 1 h, the liquid N was decanted, and the beads slowly warmed to 5 °C. Then, the beads were washed with distilled water and stored in sterilized water at 4 °C until use. Prior to use, the PVA/PEG beads were surface sterilized by washing with sodium hypochlorite and rinsed with sterile distilled water.

### Degradation of limonin by immobilized *R. fascians*: operational stability

Immobilized cells by encapsulation in the Ca-alginate hollow beads or entrapment in the PVA/PEG cryogel were placed in 100mL Erlenmeyer flasks containing 35 mL of MM medium (pH 7 and 60 µg mL^−1^limonin), synthetic citrus juice (pH 3 and 60 µg mL^−1^limonin), or natural orange juice (pH 3.5). Each flask was incubated at 25 °C on a rotary shaker (100 rpm) and the limonin content, the biomass (OD_600_) and the pH of the medium were determined every 24 h. A control set of hollow beads or PVA/PEG beads with no microbial load was also included.

Synthetic citrus juice has been previously described by Cánovas et al. (1998). This medium, with and acid pH of 3, contained KH_2_PO_4_ 0.5 mg mL^−1^, K_2_HPO_4_ 0.5 mg mL^−1^, NaCl 0.1 mg mL^−1^, NH_4_Cl 2.0 mg mL^−1^, FeCl_3_ 0.001 mg mL^−1^, MgSO_4_ 0.2 mg mL^−1^, glucose 23 mg mL^−1^, citric acid 10 mg mL^−1^, thiamine 0.001 mg mL^−1^ and limonin 60 µg mL^−1^. Fresh orange juice was obtained from Valencia late oranges by extraction through a domestic squeezer and filtered (to remove the pulp) using a glass fiber filter (type A/E, Gelman Sciences). Following Tatum and Berry (1973), samples of fresh juice were heat treated, cooled, and stored at 4 °C until use.

The operational stability of immobilized *R. fascians* under optimized conditions was assayed at pH 7 (MM medium with limonin) and at pH 3.5 (orange juice), by quantifying the reduction of limonin in consecutive cycles of repeated use. After each cycle (24 h), the hollow beads were collected, washed with CaCl_2_ (3 or 4%, w/v), and rinsed with sterilized distilled water. Likewise, the PVA/PEG beads were collected, washed with phosphate buffer (pH 8.0), and rinsed with sterilized distilled water after each cycle.

### Analytical methods

Soluble solids (ºBrix) were determined using a refractometer (Atago 3 T) according to Montenegro et al. (1995). Titratable acidity was assayed according to AOAC methods (1995) and the results were expressed as g of citric acid in 100 mL. The methods that Rosson and Chirpich (1989) and Lowry et al. (1951) have previously described for the measurement of reducer sugars and for the determination of protein content, respectively, were followed.

The limonin concentrations in MM medium and synthetic juice were spectrophotometrically measured following the method reported by Vaks and Lifshitz (1981) and modified by Martinez-Madrid et al. (1989). The determination of limonin in orange juice was carried out following the methodology proposed by Abbasi et al. (2005). A calibration curve was made with limonin between 0 and 100 ppm.

### Statistical procedure

Analysis of variance (ANOVA) was performed with the Statgraphics Centurion XVI.I software package for Windows. Results in tables and figures are expressed as the mean ± standard deviation. Significant differences in means were compared using the Tukey test at *p* < 0.05.

## Results

### Tolerance and adaptation of *R. fascians* strain to limonoids

**Fig. 1:**
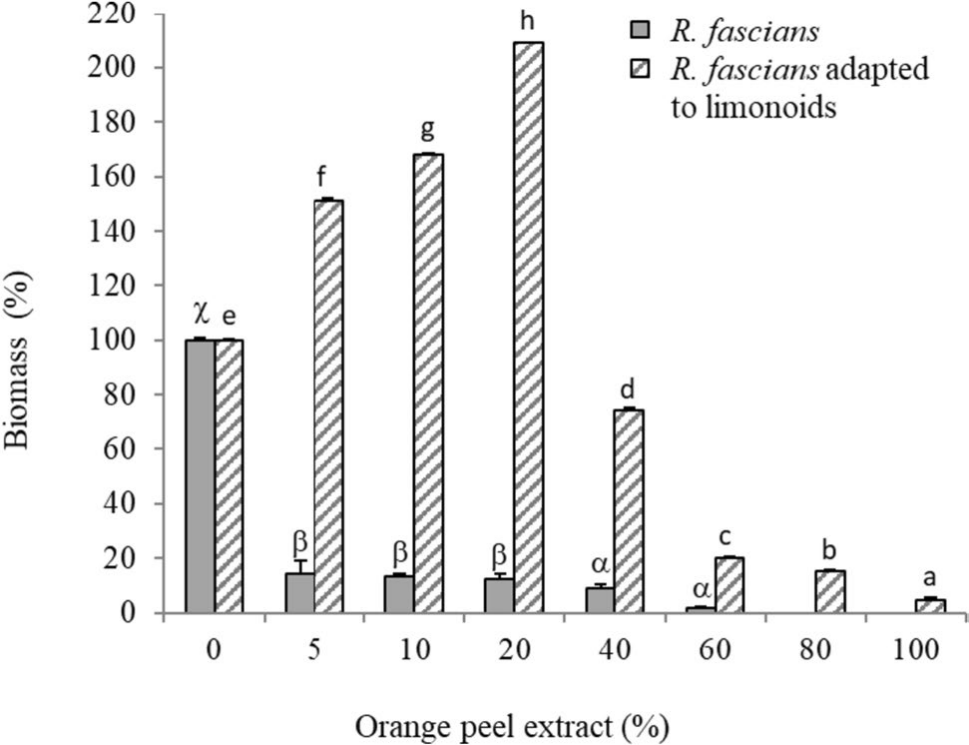
Growth of *Rhodococcus fascians* in Martínez-Madrid medium supplemented with different concentrations of orange peel extract before and after adaptation to limonoids. The biomass was determined by measuring OD_600_ after 96 h of bacterial grown. Values presented as mean (*n* = 3) and standard deviation. Latin letters (a–h) or Greek letters (α–χ) imply significant difference (for each series) according to the Tukey test (*p* < 0.05)

The tolerance of *R. fascians* to limonoids present in citrus by-products was studied by growing the microorganism in a medium supplemented with orange peel extract. The characteristics of this extract were as follows: pH 5.5, soluble solids 8.82 ºBrix, titratable acidity 4.3 mg mL^−1^ citric acid, reducing sugars 82.1 µg mL^−1^ glucose, limonoids 89.3 µg mL^−1^ limonin, and protein 12.1 mg mL^−1^. Figure 1 shows the growth of *R. fascians*, in terms of biomass production, at different concentrations of orange peel extract in the culture medium. Previously to adaptation process, a significant decrease of around 85% can be observed in microbial growth at orange peel extract concentrations between 5 and 20%. Moreover, extract concentrations higher than 60% completely inhibited the biomass production.

The adaptation procedure was made to increase the tolerance of *R. fascians* to limonoids present in citrus by-products. This adaptation of the bacterial strain was carried out by batch serial cultures with increasing concentrations of orange peel extract and subsequent agar microbial growth with limonin as only carbon source. Figure 1 shows a higher growth of the microorganism after this treatment. Specifically, at 5 and 20% orange peel extract, the microbial growth increased to 50 and 109%, respectively. Moreover, although growth rates fell at concentrations higher than 20%, at 40% orange peel extract, the biomass production was 75% versus 9% of the microorganism without previous adaptation. The microbial strain was observed to grow even at orange peel extract concentrations of 60 and 80%.

The adapted microbial strain was applied for the rest of the experiments.

### Microbial encapsulation in Ca-alginate hollow beads for limonin degradation

#### Study of limonin degradation at pH 7 (MM medium)

**Fig. 2:**
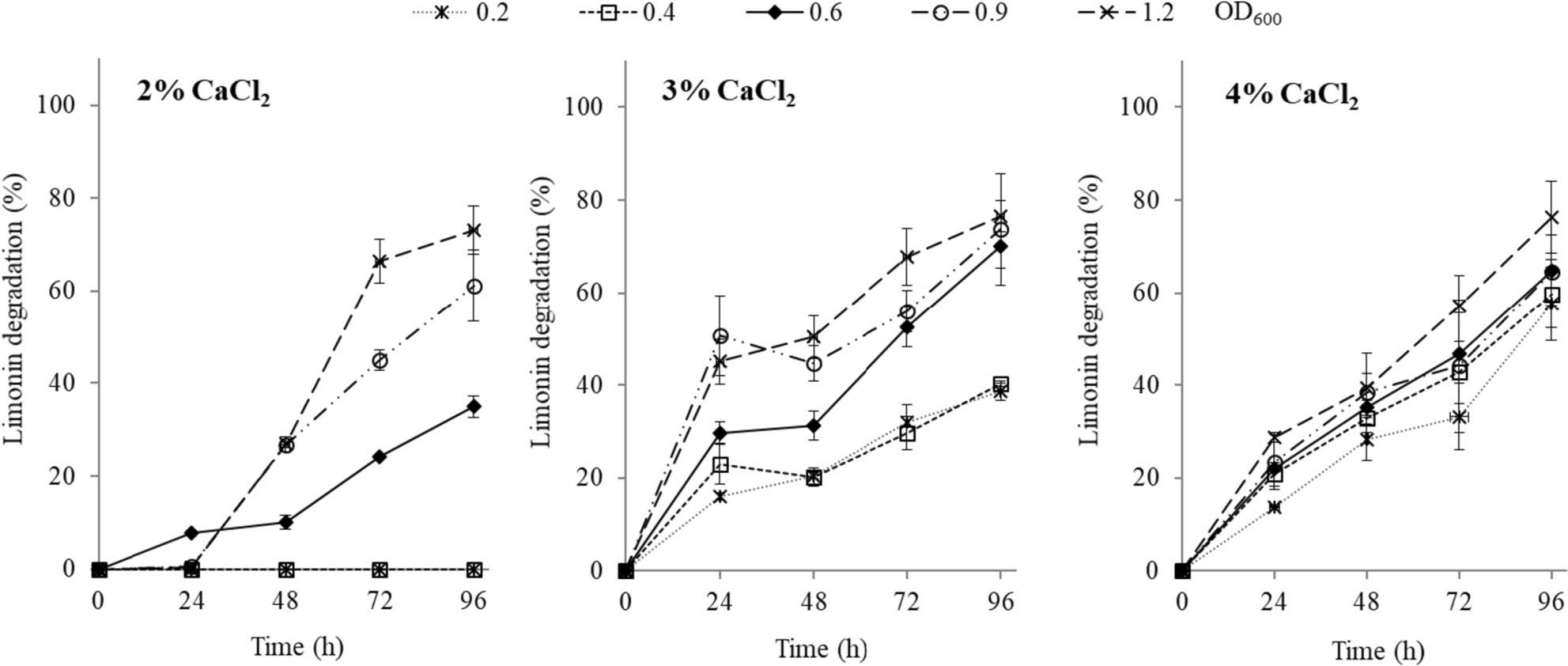
Limonin degradation, in Martinez-Madrid medium (pH 7) with limonin (60 µg mL^−1^), by *R. fascians* encapsulated in Ca-alginate hollow beads. Encapsulation was performed with 2% alginate; 4% carboxymethylcellulose (CMC); 2, 3, and 4% CaCl_2_ and different microbial load (OD_600_). Values are presented as mean (*n* = 3) and standard deviation (error bars)

To determine the optimal conditions for *Rhodococcus* encapsulation in hollow beads, the effect of CaCl_2_ concentration (2, 3, and 4%) and microbial load (OD_600_ from 0.2 to 1.2) on limonin degradation, as well as bead stability, were evaluated. In this work, 2% sodium alginate and 4% CMC were stablished based on previous studies (López-Fouz et al. 2009). The effect of CaCl_2_ concentration with different microbial loads (OD_600_ between 0.2 and 1.2) on limonin degradation is shown in Fig. 2. The increase in the concentration of CaCl_2_ for short-term treatments (24–48 h) improved limonin degradation. Thus, with 3% CaCl_2_ and 24 h, using microbial loads of 0.6, 0.9, and 1.2 OD_600_, limonin degradation of 30, 51, and 45% was found, respectively, versus 8, 1, and 1% at 2% CaCl_2_ concentration. It should also be noted that as the concentration of CaCl_2_ increased, the effect of the microbial load on limonin degradation decreased. Thus, when hollow beads were synthesized at 4% CaCl_2_, no significant differences in limonin degradation were observed with microbial loads of 0.4, 0.6, and 0.9, with values of limonin hydrolysis between 21–24%, 43–47%, and 60–65% at 24, 72, and 96 h, respectively.

**Fig. 3:**
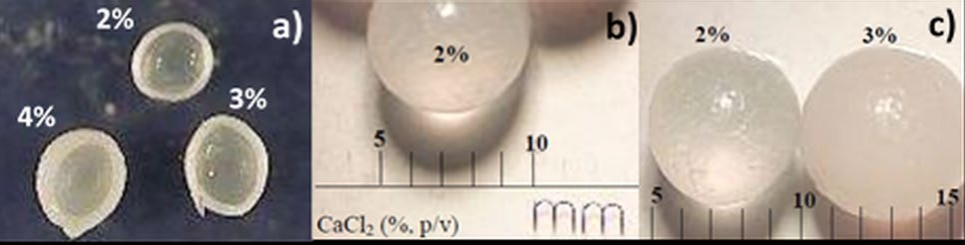
Differences in size and thickness of alginate hollow beads with the CaCl_2_ concentration (2, 3, and 4%): half-section (**a**) and whole section (**b**, **c**). Encapsulation was performed with 2% alginate and 4% carboxymethylcellulose (CMC)

Moreover, capsule size (diameter) and thickness were also evaluated at calcium concentrations of 2, 3, and 4% (Fig. 3). It was observed that both parameters (size and thickness) increased with increasing CaCl_2_ concentration.

**Fig. 4:**
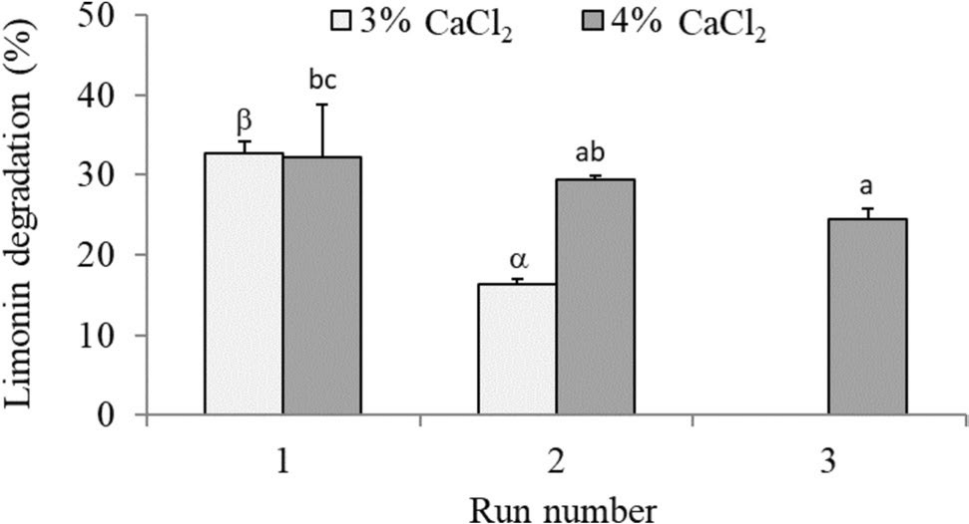
Operational stability, in Martinez-Madrid medium with limonin (60 µg mL^−1^) at 25 °C (cycles of 24 h), of *R. fascians* encapsulated in hollow beads. Hollow beads were synthesized at 2% alginate, 4% CMC, 0.6 microbial load (OD_600_) and 3 or 4% CaCl_2_. Values represent the means and vertical bars indicate standard deviation (*n* = 3). Latin letters (a–c) or Greek letters (α–β) indicate significant difference (for each series) according to the Tukey test (*p* < 0.05)

The operational stability of the microbial cells immobilized by encapsulation was also assayed. The immobilized cells were able to degrade limonin in successive cycles, resulting in a better operational stability when performing the process with 4% CaCl_2_ (Fig. 4). Specifically, the microbial load (0.6 OD_600_) encapsulated at 4% CaCl_2_ maintained the best catalytic effectiveness throughout the three successive cycles of 24 h each, with limonin degradation levels of 32, 29, and 25%, respectively.

In view of the results, hollow beads synthesized with 2% alginate, 4% CaCl_2_, 4% CMC, and a microbial load between 0.6 and 1.2 OD_600_ were selected to study the performance of immobilized cells under stress conditions (synthetic and orange juice).

#### Study of limonin degradation at acid pH (synthetic and orange juice)

**Fig. 5:**
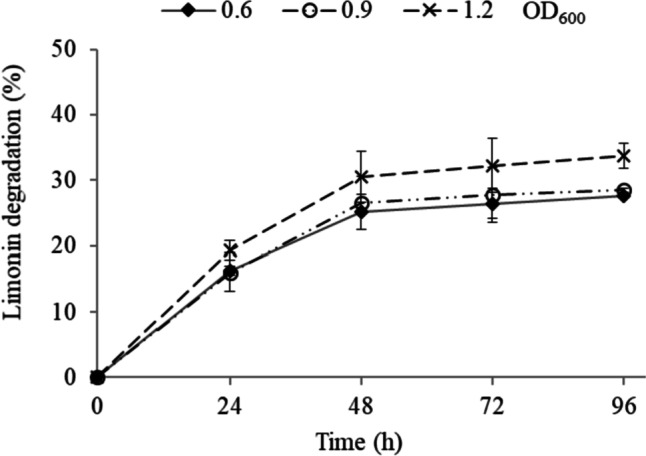
Limonin degradation, in synthetic juice (pH 3), by *R. fascians* encapsulated in Ca-alginate hollow beads. Encapsulation was performed at 2% alginate, 4% carboxymethylcellulose (CMC), 4% CaCl_2_, and different microbial loads (OD_600_). Values are presented as mean (*n* = 3) and standard deviation (error bars)

Experiments in synthetic and natural orange juice were performed in order to evaluate the effect of acid pH and citrus composition on the stability and effectiveness of the immobilized cells degrading limonin. First, experiments were performed at pH 3 and controlled composition of salts, sugars, and citric acid (synthetic juice). The results obtained are shown in Fig. 5. When *R. fascians* was immobilized by encapsulation, the highest increases in limonin hydrolysis occurred at the beginning of the treatment, with limonin degradation rates of around 16–19% and 25–31% at 24 and 48 h, respectively, depending on the microbial load. A decrease in limonin degradation of 58–56% was observed in the synthetic juice for cells encapsulated with a microbial load between 0.6 and 1.2 OD at 96 h, compared to the MM medium (pH 7). In parallel, the pH and the OD_600_ of the medium in all the samples were determined (data not shown), both factors remaining unchanged throughout the experiment. Therefore, the Ca-alginate hollow beads maintained their physical integrity in these conditions.

Finally, the operational stability in natural orange juice of the immobilized microbial cells was also assayed. The encapsulates in calcium-alginate hollow beads (immobilization conditions: 2% alginate, 4% CaCl_2_, 4% CMC, and 0.6 OD_600_) could be reused in two consecutive cycles, yielding limonin degradation levels of 18 ± 1% after the first 24 h of treatment, and 13 ± 2% after the second run of 24 h.

### Microbial entrapment in PVA/PEG cryogels for limonin degradation

#### Study of limonin degradation at pH 7 (MM medium)

**Fig. 6:**
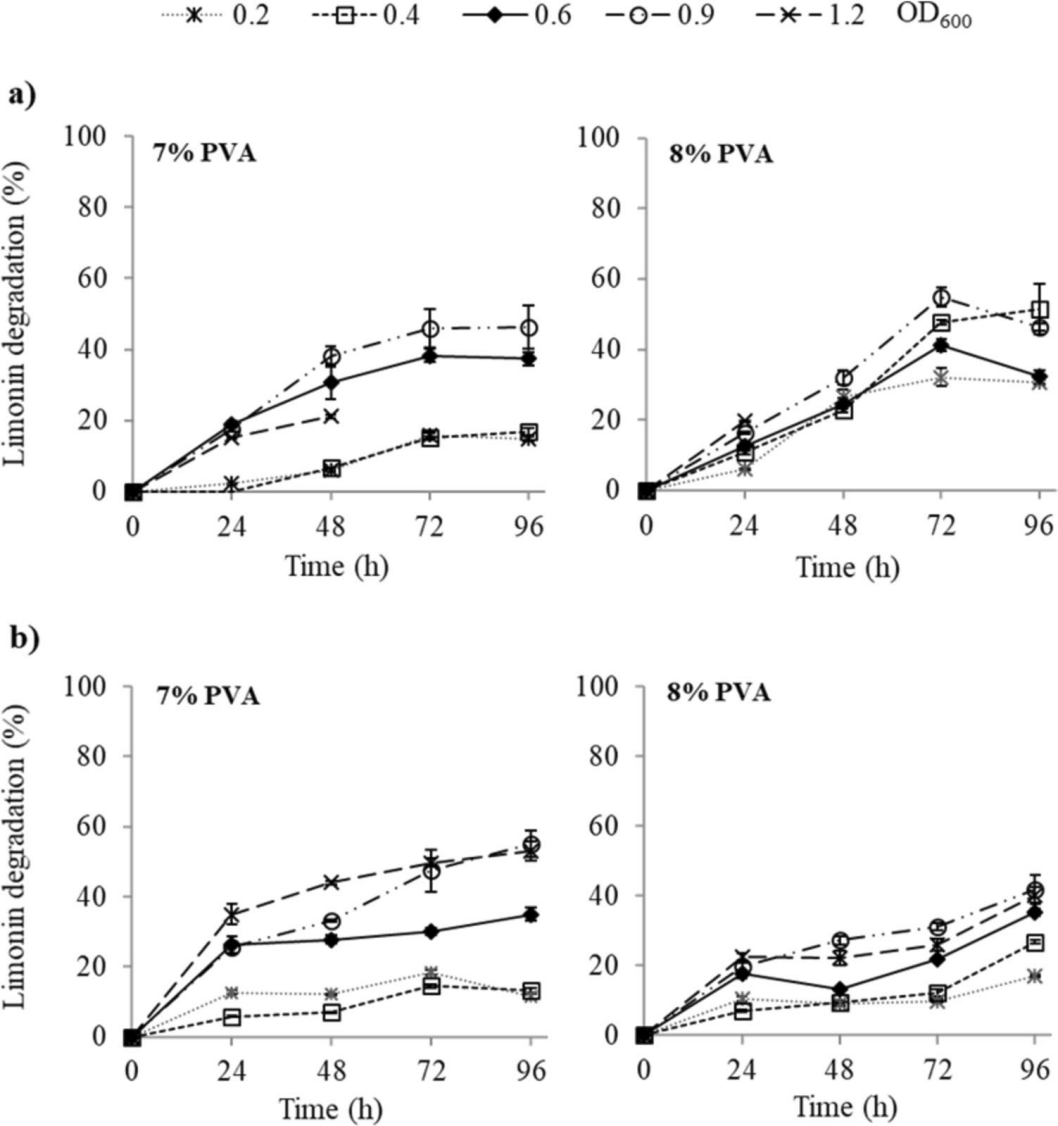
Limonin degradation, in Martinez-Madrid medium with limonin (60 µg mL^−1^), by *R. fascians* entrapped in PVA/PEG cryogels. Immobilization was carried out at 7–8% PVA, PEG concentration of 8 (**a**) and 10% (**b**), and different microbial load (OD_600_). Values are presented as mean (*n* = 3) and standard deviation (error bars)

The effect of PVA and PEG concentrations and the microbial load on limonin degradation are detailed in Fig. 6. It can be seen that at 8% PVA, the effect of the biomass incorporated in the immobilized was reduced, both for 8 and 10% PEG. The highest values of limonin degradation were found when the beads were synthesized at 8% of both PVA and PEG, reaching percentages between 32 and 55% after 72 h of treatment depending on microbial load (0.4–0.9 OD_600_). At 96 h, no appreciable increase in limonin degradation was observed.

On the other hand, high microbial loads (0.9–1.2 OD_600_) reduced the integrity of the cryogel beads with the time of treatment. For the microbial load of 1.2 OD_600_, using 8% PEG, the beads broken after 24 h in MM medium (Fig. 6). The integrity of the beads was higher at 10% PEG, and enhanced with increasing PVA concentration.

**Fig. 7:**
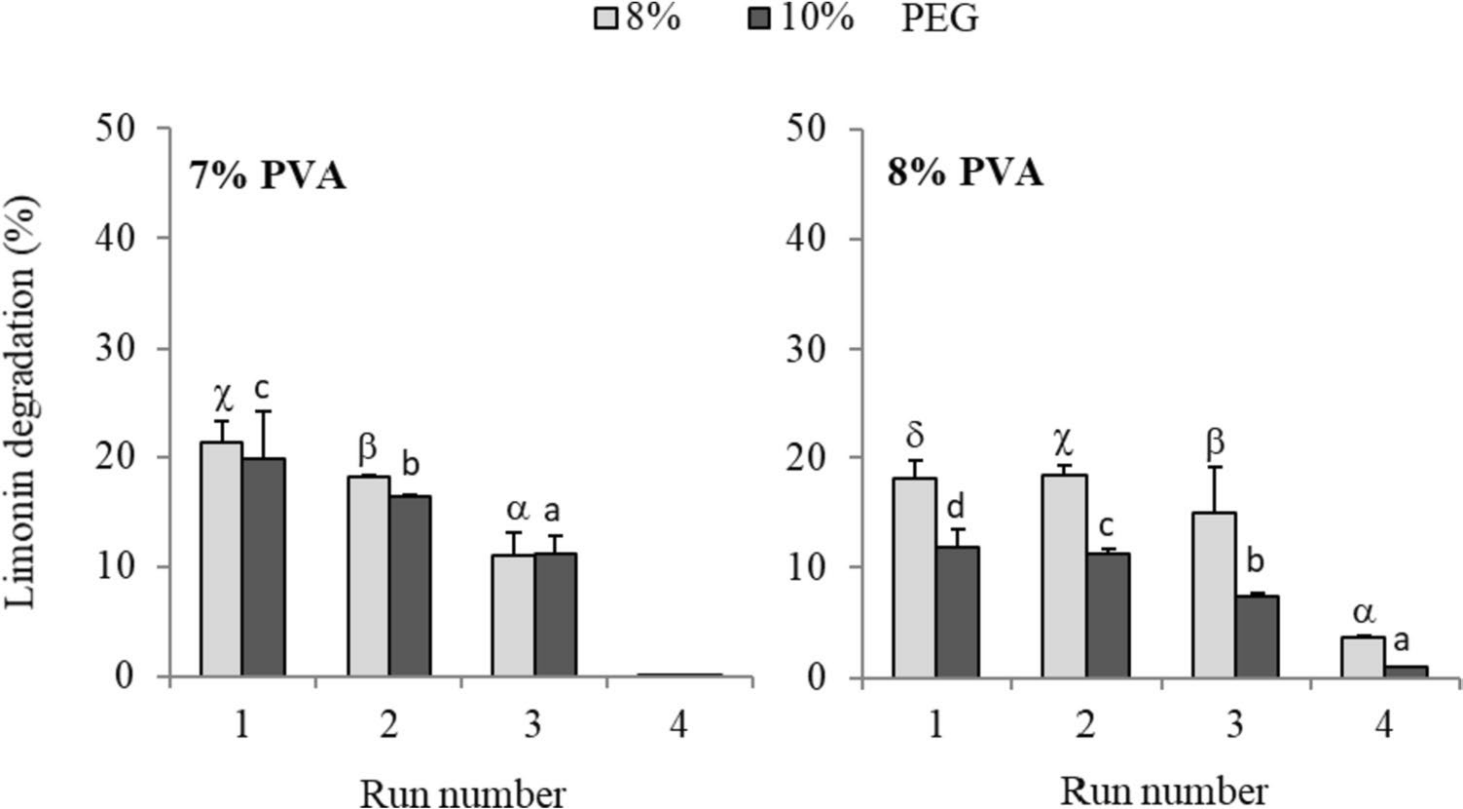
Operational stability, in Martinez-Madrid medium (pH 7) with limonin (60 µg mL^−1^) at 25 °C (cycles of 24 h), of *R. fascians* immobilized by entrapment in PVA/PEG cryogels. The entrapment was carried out at different concentrations of PVA (7 and 8%), PEG (8 and 10%), and 0.6 microbial load (OD_600_). Values represent the mean and vertical bars indicate the standard deviation (*n* = 3). Latin letters (a–d) or Greek letters (α–δ) indicate significant difference (for each series) according to the Tukey test (*p* < 0.05)

The operational stability at neutral pH (MM medium) of the entrapment cells was also assayed. While beads synthesized at 7% PVA could be reused through three cycles, those synthesized at 8% PVA withstood up to four cycles of use, with limonin degradation values of 18, 18, 15, and 4%, respectively, using 8% PEG (Fig. 7). Furthermore, limonin hydrolysis was higher with 8 than with 10% of PEG, although beads synthesized with 7% PVA showed the smallest difference in limonin degradation when changing PEG concentration.

Based on limonin hydrolysis, operation time, bead stability, and operational criteria, the most satisfactory results were obtained with 8% of both PVA and PEG, and microbial load from 0.6 OD_600_.

### Study of limonin degradation at acid pH (synthetic and orange juice)

**Fig. 8:**
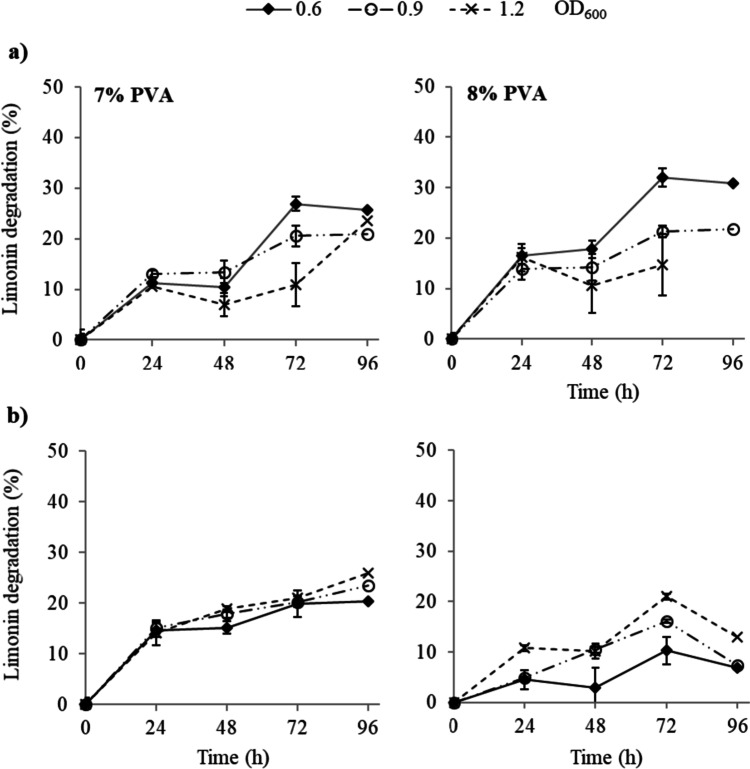
Limonin degradation, in synthetic juice (pH 3), by *R. fascians* entrapped in PVA/PEG cryogels. Immobilization was carried out at 7–8% PVA, PEG concentration of 8 (**a**) and 10% (**b**), and different microbial load (OD_600_). Values are presented as mean (*n* = 3) and standard deviation (error bars)

The effectiveness and stability of the entrapped cells in degrading limonin under stress condition were also studied. Figure 8 shows the degradation of limonin in synthetic juice by *R. fascians* immobilized in PVA/PEG cryogels. When beads were synthesized at 8% PEG (Fig. 8a), the limonin degradation was higher at 8 than 7% of PVA, although the behavior was similar throughout the experiment. At short times, no significant differences in limonin degradation were observed at the different microbial loads tested, with limonin hydrolysis values between 11 and 14–16% at 7 and 8% PVA, respectively, after 24 h of treatment. However, at long times, the higher limonin degradation corresponded to the lower microbial load assayed (0.6 OD_600_) (Fig. 8). Thus, at 72 h and 7% PVA, limonin degradation was 27 and 11% at 0.6 and 1.2 OD_600_, respectively, whereas at 8% PVA, these values reached 32 and 15%, respectively. On the other hand, the consistency of the cryogel beads improved with 10% PEG as they remained intact throughout the test, whereas 8% PEG and a high microbial load (1.2 OD_600_) resulted in bead breakage after 96 h of treatment. However, the use of 10% PEG (Fig. 8b) and 8% PVA resulted in a reduction in bitter compound degradation.

Synthetic juice stress conditions affected the catalytic action of microbial cells entrapped in the PVA cryogels, with a 22% reduction in limonin degradation (0.6 OD_600_ microbial load, 8% PVA, 8% PEG, and 72 h) compared to the MM medium at pH 7.

**Fig. 9:**
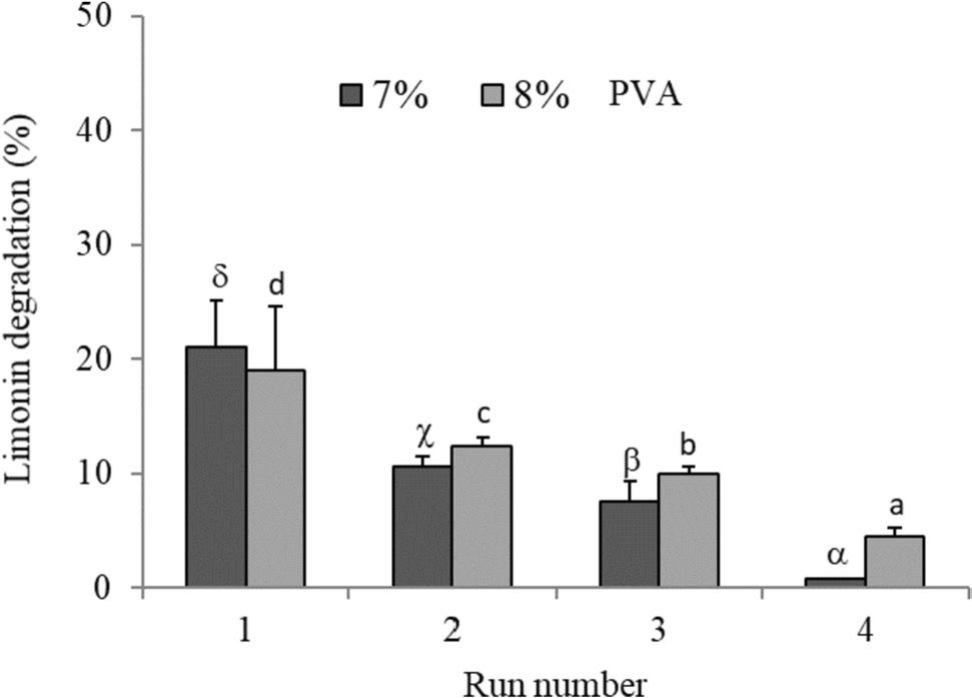
Operational stability, in orange juice (37 µg limonin mL^−1^), of *R. fascians* entrapped in PVA/PEG cryogels (8% PEG, microbial load of 0.6 OD_600_). Values are presented as mean (*n* = 3) and standard deviation (error bars). Latin letters (a–d) or Greek letters (α–δ) indicate significant difference (for each series) according to the Tukey test (*p* < 0.05)

The operational stability in orange juice of the entrapped microbial cells is shown in Fig. 9. The encapsulates in PVA/PEG cryogels could be reused through four cycles. In the first run, the limonin hydrolysis with beads synthesized at 7 and 8% PVA was 21 and 19%, respectively. After a third cycle of 24 h, the entrapped microorganisms with 7 and 8% PVA maintained limonin degradation values of 8 and 10%, respectively. After running four cycles, limonin degradation of 5% was observed by cell entrapped at 8% PVA while barely 1% was reached using PVA at 7% (Fig. 9).

## Discussion

One of the most pressing problems to consider when selecting microorganisms for industrial biotechnological applications is its resistance to the process conditions. In fact, a great part of biotechnological processes using microorganisms occurs in conditions generating stressful that negatively affect their growth and metabolism (Szymanowska-Powalowska 2015). Citrus by-products contain bioactive compounds with antimicrobial activity (Todaro et al. 2013), such as limonoids. The application of microorganisms in the treatment of citrus products and by-products requires that they be tolerant to the medium conditions. Based on this premise, a tolerance study of *R. fascians* NRRL-B-15096 to an extract from orange peel (by-product from industrial citrus processing rich in limonoids) was first carried out (Fig. 1). The growth of *R. fascians* was seriously affected by the presence of the extract in the culture medium. However, after the adaptation process, the microbial strain was able to overcome the adverse conditions and significantly increased its growth under high concentration of orange peel extract. It is well known that *Rhodococcus* strains can adapt to unfavorable conditions and respond to stress, which has been associated with the cell wall and their large array of enzymes (Pátek et al. 2021). Similar experiences for improving the adaptation of microorganisms to adverse conditions have been described by Carvalho (2012) and Tan et al. (2022).

The successful application of immobilized cells in a given industrial process largely depends on the immobilization methodology used. In this work, cells of *R. fascians* strain adapted to limonoids were immobilized by encapsulation in Ca-alginate hollow beads and by entrapment in PVA/PEG cryogels. The main differences between the two immobilization methods are that in encapsulation, the microorganisms are enclosed in hollow beads with an alginate membrane, whereas in entrapment, the cells are in the free spaces of a network composed of the synthetic polymer of PVA/PEG. The differences between both immobilization methods may affect the mass transfer and the operational stability (Lapponi et al. 2022), which in turn will determine the success of the application.

In the immobilization by encapsulation in Ca-alginate hollow beads, the activity of the encapsulated cells and the physicochemical characteristic of the hollow beads depend on various aspects such as the concentration of reactants and the cell load. In this work, the concentration of alginate (2%) and CMC (4%) was established on the basis of previous studies (López-Fouz et al. 2009). The calcium concentration affected the capsule formation mechanism, such that a higher calcium concentration resulted in a higher diameter and thickness of the capsule (Fig. 3). This effect has also been described by Chai et al. (2004). When the calcium ion solution is dripped over the alginate solution, a gel layer forms immediately around the drop. This layer grows in the axial direction of the capsule until the calcium ion contained inside the drop is consumed. In general, the gel layer formation is controlled by the diffusion of both sodium-alginate and calcium ion. However, due to volume limitation, it is fundamentally calcium that diffuses through the alginate chains and promotes gelation. Thus, as the concentration of calcium is increased, the amount of the cation per unit volume in the liquid interior of the hollow bead that is being formed is greater and, therefore, more bonds can be formed with the alginate chains, resulting in a larger size and thickness of the capsule. Furthermore, the calcium concentration could affect the rate of capsule formation, which explains why as calcium concentration increased, the effect of the microbial load in the limonin hydrolysis decreased (Fig. 2). Thus, leakage of microorganisms into the medium might have occurred during the synthesizing of the hollow beads before the alginate capsule was completely formed. This effect could be more pronounced at low concentrations of both CaCl_2_ and biomass. As an example, when the hollow beads were synthesized at 2% CaCl_2_ with low levels of microbial biomass (0.2 and 0.4 OD), microorganism loss was so high that the amount retained in the beads was insufficient to produce limonin degradation until after 96 h. At higher calcium concentration, the alginate capsule is formed more rapidly, minimizing the leakage of microbial cells. Once formed, no microorganisms were released from the hollow beads during limonin degradation, as no changes to the OD_600_ (data not shown) of the medium were observed. In addition, diffusional limitations due to the greater thickness of the capsule at 4% CaCl_2_ might explain why no differences in limonin degradation were observed among the different microbial loads that had been tested, since the calcium concentration significantly influences the permeability and the mass transfer capability of the capsule (Seifan et al. 2017). On the other hand, the higher encapsulation thickness with 4% CaCl_2_ conferred additional operational stability in MM medium (pH 7) against handling during the three successive steps, (i) bead recovery, (ii) washing, and (iii) reincorporation in a new batch, in comparison with 3% CaCl_2_ (Fig. 4).

In the immobilization by entrapment in PVA/PEG cryogels, the ratio and concentration of both PVA and PEG affect cryogel characteristics such as mechanical properties or porosity, which in turn influences bead stability and mass transfer. In a previous study using 6% of PVA (data not shown), it was observed that beads synthesized with 6% PVA had weak consistency and broke easily at microbial load above 0.6 OD_600_. Higher PVA concentration (7 and 8%) resulted in higher cryogel stability due to higher cross-linking and lower porosity and pore size (Wan et al. 2014). In addition, PEG acts as porogen agent (Xu et al. 2016) and plasticizer (Falqi et al. 2018). As porogen, PEG increase the porosity (Xu et al. 2016) and cause changes in the pore size, increasing or decreasing it depending on the PEG concentration (Taji et al. 2014), which could also affect the mass transfer. The diffusion is not only affected by the porosity, since tortuosity of the gel, generated by the immobilized cells and the matrix itself, affects mass transport (Mota et al. 2001; du Toit and Pott 2020). As plasticizer, PEG can improve the mechanical properties of cryogels (Falqi et al. 2018), which could explain the higher stability observed when beads were synthesized at 10% PEG. However, the stability of the gel structure depends on an appropriate combination of PVA and PEG. Thus, the highest efficacy and stability of entrapped cells, regardless of microbial load, were obtained with 8% PVA and 8% PEG after 72 h of treatment at pH 7 (Fig. 6).

The studies of operational stability in MM medium (pH 7) revealed that microbial cells immobilized in PVA/PEG beads withstood up to four cycles of reuse (Fig. 7) and only three in the Ca-alginate hollow beads (Fig. 4). These results indicated the higher mechanical stability against repeated use of the PVA/PEG cryogel than the hollow beads. However, the higher values of limonin degradation by the encapsulated cells in Ca-alginate hollow beads showed less diffusional limitations under these assay conditions.

Citrus by-products are characterized by an acid pH and the presence of sugars which can affect the effectivity and physical integrity of the immobilized cells. These by-products also contain certain ions, such as Na^+^ and K^+^ (Ani and Abel 2018), that can be exchanged for Ca^2+^ and can produce a continuous loss of alginate gel consistency in the hollow beads. Moreover, the affinity of anions such as citrate or phosphate for Ca^2+^ can destabilize these gels. Thus, an additional study of *R. fascians* cells immobilized on both supports in a synthetic juice medium was carried out.

A lower efficiency of limonin hydrolysis was observed in the synthetic juice (Figs. 5 and 8) than in MM medium (Figs. 2 and 6) for both entrapment and encapsulated cells, especially important for the latter. In fact, limonin degradation in the synthetic juice barely exceeded 30% for both immobilizates. These results are related to the acid pH and juice composition. Thus, the pH of the MM medium (pH 7) was close to the most favorable pH range (8.0–9.0) for the enzymatic limonin hydrolysis by *Rhodococcus* strains (Cánovas et al. 1998) while the pH of the synthetic juice was 3. However, the microenvironment of the PVA/PEG gel matrix provided better protection to the microbial cells for the degradation of limonin than that of the hollow beads against the stress conditions of the synthetic juice. The higher porosity and lower diffusion limitation of the hollow calcium alginate beads conferred less protection to the cells against acidic pH, leading to greater reduction of the degradation of the bitter compound.

The microbial load is a key factor that affects the operational stability of the immobilizates (Trevan 1990), either improving or reducing the effectiveness of the debittering process. The optimal microbial load in the immobilization process is determined by the balance between the effect on limonin degradation and the stability of the immobilizates. Excessive cell loading can lead to the breakage of the PVA/PEG beads, due to microbial growth, and the release of the cells into the medium. However, low microbial loading could result in very low levels of limonin degradation, decreasing the efficiency of the process. Szczęsna-Antczak et al. (2004) reported that the operational stability of PVA beads was strongly affected by the initial cell concentration, so that a high microbial load could cause a loss of the porous structure of the carrier. Moreover, cells immobilized by entrapment tend to grow at the periphery of the beads, which can lead to gel surface breakage and cell leakage when beads are synthesized with high microbial load (Koyama and Seki 2004). In our experience, synthesis of both Ca-alginate hollow beads and the PVA/PEG cryogel beads at microbial loads higher than 0.6 OD_600_ hindered the immobilization procedure, and generally did no led to further limonin reduction in the synthetic juice, so 0.6 OD_600_ was selected as the most suitable microbial load.

Finally, the operational stability in orange juice of *R. fascians* cells immobilized by both methods under the optimized conditions was investigated. The entrapped cells in PVA/PEG cryogels were more stable in the natural juice than the encapsulated in calcium alginate. Thus, those immobilized in cryogels allowed up to four 24-h reuse cycles, while those encapsulated in calcium alginate allowed only two cycles. Limonin degradation was also higher for entrapped cells than for encapsulated cells. Limonin degradation remained in the same range compared to synthetic juice for both immobilization procedures, indicating that the composition of a natural citrus product does not introduce additional elements that would imply a reduction in the degradation of the bitter compound.

In conclusion, the effectiveness of *R. fascians* cells immobilized by both encapsulation and entrapment to degrade limonin under the acidic pH and compositional conditions of a citrus product was tested. In general, cells entrapped in PVA/PEG gels showed greater stability and efficiency in degrading limonin than cells encapsulated in Ca-alginate hollow beads. Therefore, the application of immobilized microbial cells for the debittering of citrus by-products could be a feasible alternative to chemical treatments to reduce the loss of bioactive compounds in the by-products, and also to preserve the environment. However, further research is needed in this field, such as the use of continuous processes as an alternative to batch bioreactors to reduce mechanical damage to the immobilized biocatalysts in each reuse cycle and to achieve even higher yields of bitter compound degradation.

## Data Availability

The datasets generated during and/or analyzed during the current study are available from the corresponding author on reasonable request.
